# Receptor Plants Alleviated Allelopathic Stress from Invasive *Chenopodium ambrosioides* L. by Upregulating the Production and Autophagy of Their Root Border Cells

**DOI:** 10.3390/plants12223810

**Published:** 2023-11-09

**Authors:** Qiang Wang, Xijie Zhou, Shengli He, Wenguo Wang, Danwei Ma, Yu Wang, Hong Zhang

**Affiliations:** 1 College of Life Science, Sichuan Normal University, Chengdu 610101, China; wang_cunxin@126.com (Q.W.); zhouxj0721@163.com (X.Z.); ldsheshengli@126.com (S.H.); wangyu_765@163.com (Y.W.); 13678021901@163.com (H.Z.); 2 Key Laboratory of Development and Application of Rural Renewable Energy, Biogas Institute of Ministry of Agriculture and Rural Affairs, Chengdu 610041, China

**Keywords:** *Chenopodium ambrosioides* L., allelopathic stress, root border cells, release, autophagy

## Abstract

*Chenopodium ambrosioides* L. is an invasive plant native to the Neotropics that has seriously threatened the ecological security of China, and allelopathy is one of the mechanisms underlying its successful invasion. Maize (*Zea mays* L.) and soybean (*Glycine max* (L.) Merr.), as the main food crops, are usually affected by *C. ambrosioides* in their planting areas. The purpose of this study was to investigate the ultrastructure, autophagy, and release-related gene expression of receptor plant root border cells (RBCs) after exposure to volatile oil from *C. ambrosioides* and its main component α-terpene, which were studied using maize and soybean as receptor plants. The volatiles inhibited root growth and promoted a brief increase in the number of RBCs. As the volatile concentration increased, the organelles in RBCs were gradually destroyed, and intracellular autophagosomes were produced and continuously increased in number. Transcriptomic analysis revealed that genes involved in the synthesis of the plasma membrane and cell wall components in receptor root cells were significantly up-regulated, particularly those related to cell wall polysaccharide synthesis. Meanwhile, polygalacturonase and pectin methylesterases (PME) exhibited up-regulated expression, and PME activity also increased. The contribution of α-terpene to this allelopathic effect of *C. ambrosioides* volatile oil exceeded 70%. Based on these results, receptor plant root tips may increase the synthesis of cell wall substances while degrading the intercellular layer, accelerating the generation and release of RBCs. Meanwhile, their cells survived through autophagy of RBCs, indicating the key role of RBCs in alleviating allelopathic stress from *C. ambrosioides* volatiles.

## 1. Introduction

Allelopathy is a phenomenon in which one plant affects the growth, survival, development, and reproduction of another plant through the release of allelochemicals [1], and the negative effects of allelopathy are commonly referred to as allelopathic stress [2]. Allelochemicals are a class of biologically active secondary metabolites, including simple hydrocarbons and complex polycyclic aromatic compounds, such as phenols, tannins, steroids, terpenoids, glycosteroids, and alkaloids [3,4]. The release pathways of allelochemicals include volatilization, rain-fog leaching, root secretion, and decomposition of residual plant matter [5,6]. The release of allelochemicals by either pathway results in them entering the soil and affecting the root growth of surrounding plants. *Chenopodium ambrosioides* L. (Chenopodiaceae) is an annual or perennial aromatic herb native to the Neotropics [7]. It has now spread to most parts of China and become one of the invasive plant species threatening the ecological security of China [8,9]. There is ample evidence that the invasiveness of invasive plant species can involve suppression of the growth and phenotypic traits of native plants [10]. Inhibition of the growth of surrounding plants by the release of volatile allelochemicals has been shown to be the main mechanism of successful invasion of *C. ambrosioides* [11,12]. The whole plant is rich in volatile compounds, and more than 20 compounds have been identified in the volatile oil of *C. ambrosioides*, among which α-terpinene is a common component in different populations and has been shown by the research group to be the primary allelochemical of *C. ambrosioides* [13,14]. It has also been shown that α-terpinene can become fixed in soil ecosystems by adsorption [10].

Plant survival depends on the ability of root tips to perceive and absorb soil water and nutrients [15]. Root cap cells differentiate to produce root border cells (RBCs), which have protective functions for the root tip [16,17,18]. RBCs secrete a variety of metabolites outward to form an extracellular mucilage layer, which together forms the root extracellular trap (RET) structure, constituting the first active soil–plant interface and playing an important role in root tip stress resistance [19]. As a “defense cell”, the number and activity of RBCs are closely related to their function of protecting the root tip [20]. In recent years, RBCs have emerged as a single-cell model system for studying environmental stresses, including allelopathic stress [21]. Under adversity, plants can produce more RBCs [22,23], which secrete substances such as arabinogalactan proteins (FLAs) extracellularly [24]. These substances can thicken the extracellular mucilage layer and expand the RET to sequester exogenous heavy metals and allelochemicals [25] and even prevent the invasion of pathogenic bacteria [26,27,28]. When the RET is disrupted, the cytotoxicity of allelochemicals can lead to a burst of reactive oxygen species (ROS) in the receptor plant root, thereby activating the oxidative stress response and causing cell damage [29]. Our previous study found that the viabilities of RBCs significantly decrease with exposure to *C. ambrosioides* volatile oil and its major component α-terpinene. The volatile oil from *C. ambrosioides* had the potential to induce the enlargement of RET in a dose-dependent manner. In addition, we also found that degradation of exDNA or extracellular proteins with DNase I or protease resulted in a loss of the resistance of RBCs to allelochemical stress and a decrease in the RET area [30]. Under the action of volatile oil from *C. ambrosioides*, the contents of both ROS and nitric oxide (NO) in the root tips increase [31]. Further research found that the levels of ROS and NO in RBCs increased in a concentration-dependent manner. Analysis showed a significant positive correlation between cell mortality and ROS and NO levels. The addition of exogenous ROS scavengers’ ascorbic acid (AsA), nitrate reductase inhibitors (NaN_3_), and pan Caspase inhibitors ZVADFMK can effectively alleviate the cell lethal effect of volatile substances [32].

Autophagy is a widespread phenomenon in eukaryotes that degrades proteins and organelles to facilitate the recycling of cellular materials [33]. During plant growth and development, autophagy is continuously maintained to ensure homeostasis of the internal environment at basal levels. The up-regulation of autophagy is generally beneficial to plant survival and improves plant tolerance to abiotic stresses such as heat, salt, and drought under environmental stress [34]. ROS are important signaling molecules that regulate apoptosis. Additionally, they can induce cellular autophagy by activating the MCOLN1-lysosomal Ca^2+^-TFEB pathway [35,36], which promotes the clearance of damaged mitochondria and excess ROS to effectively promote cell survival [37,38].

In light of these scientific data, RBCs play an important role in the root tip. However, some key questions still have unclear answers. How does the receptor root system regulate the release of RBCs under the allelopathic stress induced by *C. ambrosioides*? Do RBCs survive through autophagy against chemotactic stress from *C. ambrosioides*? Answering these questions will help elucidate the molecular ecological processes of *C. ambrosioides* allelopathy. Therefore, in this study, we analyzed the effects of *C. ambrosioides* volatile oil and its primary allelochemical, α-terpinene, on the formation, release, and autophagy of receptor plant RBCs and their mechanisms using submicroscopic techniques, laser confocal scanning microscopy, and transcriptomic techniques. By using *Zea mays* L. (exotic species) and *Glycine max* (L.) (native species), which are crops that are commonly grown under invasion by *C. ambrosioides*, as receptor plants, the aim was to analyze the resistance of RBCs to the volatiles of *C. ambrosioides* at the microscopic, submicroscopic, and molecular levels as well as by using the adjustment strategies that root tips may exert on the production and release of RBCs during this process.

## 2. Materials and Methods

### 2.1. Materials

The test material (*C. ambrosioides*) was harvested in November 2019 from Jianyang City, Chengdu, Sichuan Province, China (N 30°38′7″ E 104°34′55″). It was collected at the reproductive stage from plants with good growth, cut into small sections of about 2 cm in length, and dried in the shade. The volatile oil was extracted by hydrodistillation [39], and each 1.5 kg of *C. ambrosioides* material was distilled for 3 h, dried by adding anhydrous Na_2_SO_4_, and then stored in a refrigerator at 4 °C. The relative percentage activity of α-terpinene in this volatile oil was measured as 45.03% using an Agilent 7890A/5975c gas chromatography-mass spectrometer (GC-MS) (Agilent Technologies, Santa Clara, CA, USA) and chromatographic peak area normalization [40].

The standard α-terpinene used in the test was purchased from Shanghai Aladdin Reagent Company (≥90%) (Shanghai, China).

The receptor plant seeds of *Z. mays* (‘Zhengdan 958’) and *G*. *max* (‘Tiedou 80’) were purchased from Shouguang Jiahe Seed Industry Co., Ltd. (Qingdao, China) and Chengdu Jinjiang District Fengzhi Seed Industry Co. (Chengdu, China), respectively.

### 2.2. Material Cultivation

The cultivation of *Z. mays* and *G*. *max* seeds used the pure agar medium suspension air method [41]. Pure agar medium was prepared by pouring 30 mL of 0.7% pure agar into glass culture flasks (90 mm high, 68 mm in diameter). Seeds were selected with full particles, uniform size, and no insect spots and sterilized with 0.5% potassium permanganate solution (KMnO_4_) for 8 min, washed in distilled water, soaked in water until fully swollen (24 h for *Z. mays* and 6 h for *G max*), and then evenly spread on enamel plates lined with a double layer of moistened gauze, after which they were further incubated until they germinated. Imbibition and culture were performed in an incubator at 25 °C in the dark; after the radicles appeared, the seeds were placed upside down in the medium (10 seeds/bottle for *Z. mays* and 5 seeds/bottle for *G*. *max*) and set aside.

### 2.3. RBC Removal Assay

After 12 h of cultivation, the RBCs were removed every 6 h according to a slight modification of the method used by Huskey et al. [25], (recorded as group N); the root tip was immersed in distilled water for 2 min to disperse the RBCs and extracellular secretions, and then they were gently scraped onto gauze. Some plants with RBCs were retained as the control group (recorded as group W). The volatile oil treatment group (V) seeds were exposed to concentration gradients of V1 (6.25 μL/L), V2 (12.50 μL/L), and V3 (25.00 μL/L). The α-terpinene contents of T1 (3.125 μL/L), T2 (6.255 μL/L), and T3 (12.510 μL/L) were set according to the V1, V2, and V3 concentration gradient. The volatiles of different concentration gradients were added as drops to the caps of the culture flasks, respectively. The caps were screwed tight, with the treatment without volatiles as the control group (CK). All groups were incubated in an inverted incubator under incubation conditions of 25 °C and darkness. Parameters were measured at the end of treatment.

#### 2.3.1. Root Length Determination

Receptor plant roots (5 from *Z. mays* and 3 from *G. max*) were harvested at 6, 12, and 24 h after treatment was initiated, and the lengths were measured with a ruler, with each treatment replicated three times.

#### 2.3.2. Root Extracellular Trap Area Determination

The RET area was determined according to the method of Xiao et al. [42]. After 24 h of treatment, the root tips were placed on a glass slide. Then, 10 μL of India ink solution (ddH_2_O: India ink = 5:1) was added to the root tip area and stained for 90 s. The RET area was observed and photographed using a Nikon ECLIPSE 55i microscope.

#### 2.3.3. Root Cap Pectin Methyl Esterase Activity Assay

Root cap pectin methyl esterase (PME) activity was determined according to the method of Richard et al. [43] after 6, 12, and 24 h of treatment, respectively. In brief, 0.01 mol/L hydrochloric acid solution was mixed with PME substrate solution, and the absorbance value was measured at 525 nm after 2 h in a water bath at 37 °C to establish a standard curve; root tips of approximately 3 mm in length from W groups (5 from *Z. mays* and 3 from *G. max*) were randomly cut, 200 μL of PME extract (0.2 mol/L Na_2_HPO_4_, 0.1 mol/L citric acid, 1 mol/L NaCl, pH 5.8) was ground in an ice bath, and the supernatant was collected by centrifugation at 13,000 rpm for 10 min at 4 °C. Absorbance was measured at 525 nm, and the activity was calculated based on the standard curve in μmol H^+^root cap^−1^·h^−1^. Each treatment was repeated in triplicate.

#### 2.3.4. RBC Count Determination

After 2, 4, 6, 12, and 24 h of treatment, root tips approximately 5 mm long were cut and immersed in 200 μL of distilled water (5 tips/tube for *Z. mays* and 3 tips/tube for *G. max*) and shaken on a vortex shaker for approximately 30 s to obtain 200 μL of receptor RBC suspension. Then, 100 μL of 0.4% trypan blue dye was added for 1 min; cells on cell counting plants were counted, and each treatment was repeated five times.

#### 2.3.5. Transcriptome Analysis

After 24 h of treatment, referring to the method of Fatima et al. [44], the root tips of the control group, V2, and T2 treatment groups were immersed in liquid nitrogen and promptly ground. Total root tip RNA was extracted with Trizol solution using standard extraction methods. RNA purity was checked using a NanoPhotometer^®^ spectrophotometer (Implemen, Westlake Village, CA, USA), and RNA integrity was assessed using an RNA Nano 6000 assay kit (Bioanalyzer 2100 system, Agilent Technologies). RNA with integrity values greater than 8.0 was used for sequencing library construction, and the libraries were sequenced for transcriptomic analysis on the Illumina HiSeq 4000 platform (Illumina, San Diego, CA, USA). The unigenes were annotated according to several publicly available protein databases, including the NCBI non-redundant protein sequences (NR), Gene Ontology (GO), Kyoto Encyclopedia of Genes and Genome (KEGG), Evolutionary Genealogy of Genes: Non-supervised Orthologous Groups (eggNOG), Protein family (Pfam), Swiss-Prot protein Sequence (Swiss-Prot) databases. Differential gene expression and pathway analysis were performed using the DESeq2 R package (1.16.1), with *p*_adj_ < 0.05 and |log_2_(fold change)| > 1 as the threshold criteria for identifying differences. GO enrichment and KEGG pathway analysis were implemented to determine which differentially expressed genes (DEGs) were in significantly enriched GO terms and metabolic pathways at *p*_adj_ < 0.05, using the topGO R packages and KOBAS software (http://www.genome.jp/kegg/, accessed on 1 December 2021), to analyze post-stress transcript differences between treatment groups (Table 1).

Two micrograms of RNA were reverse-transcribed into cDNA. Primers (Table S1) were designed using the NCBI website [Primer designing tool (https://www.ncbi.nlm.nih.gov/tools/primer-blast/, accessed on 18 March 2022)]. Amplification was conducted with the MiniOpticon™ Real-Time PCR Detection System (Bio-Rad, Hercules, CA, USA), Bio-Rad CFX Manager (V 3.1) Real-Time Sequence Detection System, and 2×ChamQ Universal SYBR qPCR Mix Kit (Novozymes, Nanjing, China) for RT-qPCR. The *actin*-1 and *β-Actin* genes were used as internal reference genes in *Z. mays* and *G. max*, respectively [45,46]. The 2^−ΔΔCt^ method was used to analyze differential expression. Each treatment included three biological replicates, and each sample included three technical replicates.

### 2.4. Cytotoxicity

After the receptor plants were cultured until their roots were 20–30 mm long, root tips that were approximately 5 mm long were cut and immersed in 200 μL of distilled water (5 tips/tube for *Z. mays* and 3 tips/tube for *G. max*) and shaken on a vortex shaker for approximately 30 s to obtain 200 μL of RBC suspension of the receptor roots. The volatile oil treatment master mix (0.1 μL/μL) and α-terpinene master mix (0.05 μL/μL) were prepared with 25% dimethyl sulfoxide (DMSO). Five treatment gradients were established with 1, 2, 3, 4, and 5 μL of treatment master mix volumes made up to 5 μL with 25% DMSO, added to the RBC suspension, mixed well, and incubated in an incubator at 25 °C for 30 min in the dark with 5 μL of distilled water as control (CK), and each treatment was repeated three times.

#### 2.4.1. ROS Content Measurement

After treatment, the RBC suspensions were centrifuged (4 °C, 1000 rpm) for 5 min, the supernatant was discarded, and the precipitate was rinsed twice in PBS buffer. DCFH-DA solution from the DCFH-DA Fluorescent Probe Kit (Beyoncé, Shanghai, China) was diluted to 10 μM, and 100 μL was added to the precipitate, which was then incubated at 37 °C for 30 min without light. The RBCs were centrifuged (4 °C, 1000 rpm) for 5 min, and the supernatant was discarded, washed three times with PBS, and resuspended in 200 μL of PBS. Absorbance values were measured using a SpectraMax M2 ELISA (Molecular Devices, San Jose, CA, USA) at an excitation wavelength of 488 nm and an emission wavelength of 525 nm, and each treatment was repeated five times. Relative fluorescence values were calculated using the control group as the baseline value.

#### 2.4.2. RBC Activity Assay

RBC activity was determined according to the method of Ma et al. [29]. At the end of the treatment, after 20 μL of cell suspension was collected from each sample, 8 μL of AO/EB staining solution (1/1, *v*/*v*) was added and mixed, and samples were stained for 1 min under protection from light. Samples were then observed with a Nikon ECLIPSE 55i fluorescence microscope (Nikon, Tokyo, Japan) and photographed. Each treatment was repeated three times. The number of dead cells and the total number of cells were counted, and cell mortality was calculated as follows: Cell mortality rate = number of dead cells/total number of cells × 100%.

#### 2.4.3. Ultrastructural Observation of RBCs

To observe the ultrastructure of RBCs, 200 μL volumes of the RBC suspensions were collected from the treated groups (4 μL treatment master mix groups) and control groups and centrifuged at 12,000 rpm for 10 min. The supernatant was discarded, and the precipitate was retained, pre-fixed with 3% glutaraldehyde, post-fixed with 1% osmium tetroxide, dehydrated in graded acetone, and embedded in Ep812. After preparing ultra-thin slices with a thickness of about 60–70 nm using an ultramicrotome EM UC 7 (Leica, Wetzlar, Germany), they were floated on the liquid surface of the knife slot and then fished out onto a copper mesh. The resulting semi-thin sections were stained with toluidine blue for optical localization. Then, ultrathin sections were cut with a diamond knife, and uranyl acetate and lead citrate were stained for observation with a JEM-1400FLASH transmission electron microscope (JEOL, Tokyo, Japan).

#### 2.4.4. Autophagy Detection

Autophagic vesicle assay: After treatment, 200 μL RBC suspensions from the control group and treated groups (4 μL treatment master mix groups) were collected, centrifuged (4 °C, 1000 rpm) for 5 min, and rinsed once with PBS, and 100 μL of the resulting supernatant was discarded. Then, 20 μL of Dansylcadaverine (MDC) staining solution was added to the remaining RBC suspension according to the instructions of the MDC Fluorescent Probe Kit (Source Leaf, Shanghai, China), gently mixed, and incubated at 37 °C. The RBCs were incubated at 37 °C for 30 min, rinsed twice with PBS, and observed and photographed by laser confocal microscope (Olympus SpinSR10, Tokyo, Japan) (excitation wavelength 355 nm, blocking wavelength 512 nm).

Autophagy Inhibition Assay: The autophagy inhibitor 3-methyladenine (3-MA) from the 3-MA kit (Source Leaf, Shanghai, China) was prepared in 10 mmol/L and 20 mmol/L solutions. Root tips approximately 5 mm long were cut and immersed in 200 μL of the two different concentrations of 3-MA solution. Then, 5 μL of essential oil and the corresponding concentration of α-terpinene (4 μL treatment master mix groups) were added to the suspension, and 5 μL ddH_2_O was added to the control group. After incubation at 25 °C for 30 min in a dark incubator, cell activity was detected by staining with AO/EB staining solution for 1 min as described above, and three replicates of each treatment were observed.

### 2.5. Statistical Analysis

One-way analysis of variance (ANOVA), Tukey’s test, and multiple comparison tests (LSD) were performed using SPSS 25.0 (IBM Corp., Armonk, NY, USA) and Excel 2019 (Microsoft Corp., Redmond, WA, USA), and GraphPad Prism 9 (GraphPad Software, Boston, MA, USA) was used to make graphs. Bioinformatic analysis was performed with the OmicStudio tool (https://www.omicstudio.cn/tool, accessed on 15 November 2022).

## 3. Results and Analysis

### 3.1. Effect of RBCs on the Growth of Receptor Roots Induced by the Action of Volatiles from C. ambrosioides

**Figure 1:**
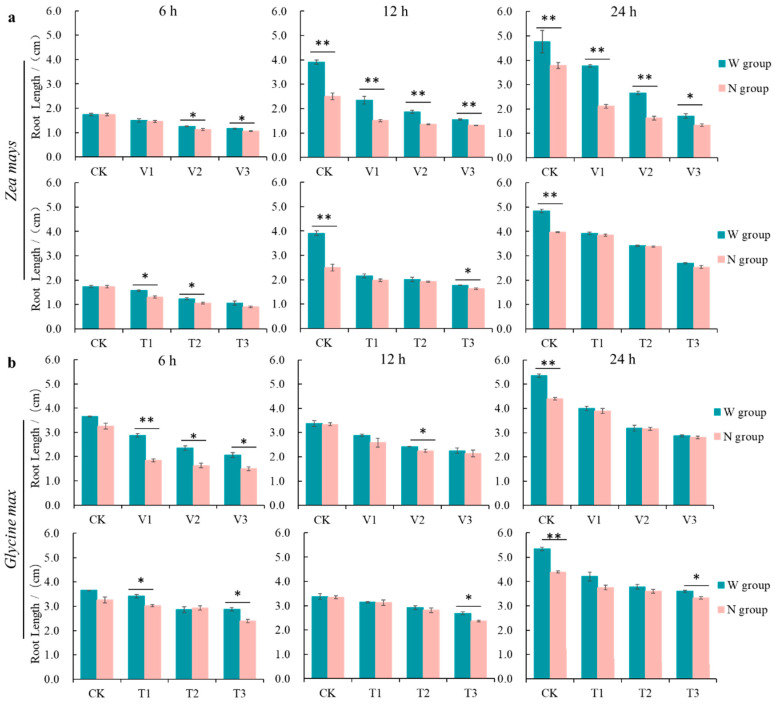
Effects of root border cell (RBC) retention and removal on the root length of *Zea mays* and *Glycine max* under the stress of volatile oil and α-terpinene from *Chenopodium ambrosioides*. (**a**) *Zea mays* receptor. (**b**) *Glycine max* receptor. Note: W, retention of RBCs; N, removal of RBCs; the volatile oil treatment group (V): V1 (6.25 μL/L), V2 (12.50 μL/L), and V3 (25.00 μL/L). The α-terpinene treatment group (T): T1 (3.125 μL/L), T2 (6.255 μL/L), and T3 (12.510 μL/L), the same below. *, significantly different; **, highly significantly. *p* < 0.05.

The growth of the receptor roots was inhibited by the volatiles of *C. ambrosioides* (Figure 1). Among the two receptor plant species growth phenotypes, the root length of *Z. mays* showed a concentration-dependent decrease, while the root growth of *G. max* was inhibited to an extent that showed both time- and concentration-dependent effects. The degree of inhibition was more pronounced in the RBC removal treatment group compared to the RBC retention group. At the time of maximum root growth inhibition, the root lengths of the *Z. mays* and *G. max* RBC retention treatment groups were 1.78 and 1.56 times higher than those of the removal treatment group, respectively. At this same time point, the root lengths of the control *Z. mays* and *G. max* control groups were 2.82 and 2.20 times higher than those of the volatile oil treatment group, respectively, and 2.21 and 1.49 times higher than those of the α-terpinene treatment group, respectively. The chemosensory potential of the α-terpinene treatment group of *Z*. *mays* and *G*. *max* were 0.78 and 0.68 times higher than those of the volatile oil treatment group, which played an important role in the chemosensory stress caused by *C. ambrosioides*. These results suggest that *C. ambrosioides* volatile oil and α-terpinene inhibited the growth of the receptor roots, while the RBCs had a mitigating effect on the chemosensory stress.

### 3.2. Changes of Root Extracellular Trap Area in the Receptor Plant by Treatment with C. ambrosioides Volatiles

**Figure 2:**
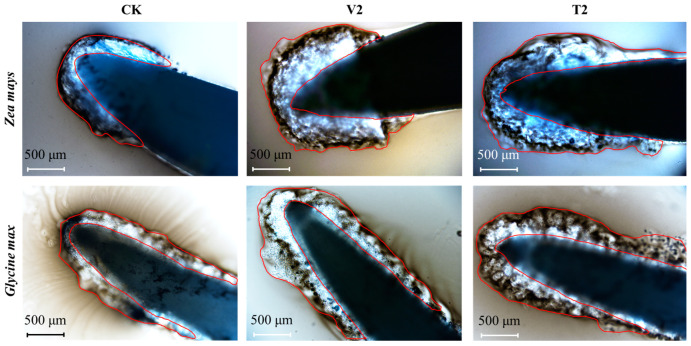
Root extracellular trap (RET) micrograph of *Zea mays* and *Glycine max* under the stress of volatile oil and α-terpinene from *Chenopodium ambrosioides*. Note: The red range is the RET. The volatile oil treatment group (V): V2 (12.50 μL/L). The α-terpinene treatment group (T): T2 (6.255 μL/L).

The RET area was affected by the volatiles of *C. ambrosioides* (Figure 2). After staining with Indian ink solution, RET accumulated in the anterior part of the root tip in the control group, and mucus components accumulated without dispersion. After treatment with a V2 concentration of volatile oil, the area of RET increased compared to the control group. Among them, the area of RET in *G. max* roots showed the most significant change, and the extracellular mucilage layer was partially released and dispersed outward. At the T2 concentration of α-terpinene, the area of RET in the roots of both receptors also increased, and the extracellular mucus components were dispersed to varying degrees.

### 3.3. Changes of PME Activity in the Receptor Plant by Treatment with C. ambrosioides Volatiles

**Figure 3:**
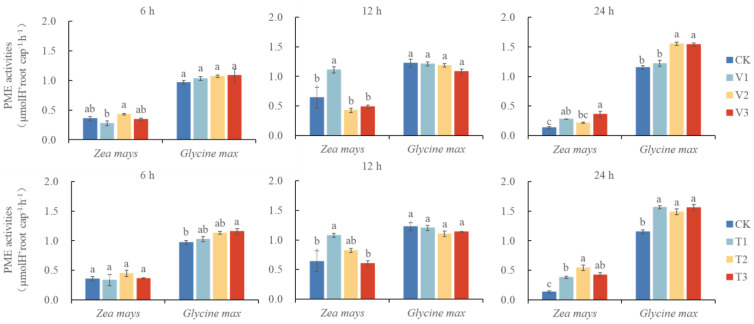
Changes in pectin methylesterase activity in the root tip of two receptor plants under the stress of volatile oil and α-terpinene from *Chenopodium ambrosioides.* Note: different lowercase letters indicate the difference significant at the 0.05 level, the same below.

*C. ambrosioides* volatiles promoted receptor root cap PME activity (Figure 3). The root cap PME activities of the two receptor species responded differently to *C. ambrosioides* volatile oil treatment, with *Z. mays* showing an overall dual time-concentration-dependent effect and *G. max* showing a time-dependent increase, with more pronounced changes in the 24 h treatment group. At the highest root cap PME activity levels in *Z. mays* and *G. max*, that of the volatile oil treatment group was 2.73 and 1.35 times higher than that of the control group, respectively, and that of the α-terpinene treatment group was 4.10 and 1.36 times higher than that of the control group, respectively. The α-terpinene treatment group had 1.50 and 1.01 times higher chemosensitization potential than the volatile oil treatment group for *Z. mays* and *G. max*, respectively. Thus, volatile oil and α-terpinene induced increased root cap PME activity in the receptor root cap, which could accelerate the release of RBCs, and α-terpinene had an important role in the chemosensory stress induced by volatile oil from *C. ambrosioides*.

### 3.4. Changes in the Number of Receptor RBCs Induced by Allelopathic Stress from C. ambrosioides

**Figure 4:**
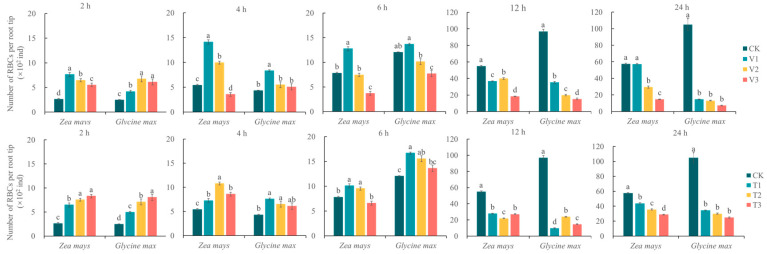
Changes of the root border cell quantity of two receptor plant species under the stress of volatile oil and α-terpinene from *Chenopodium ambrosioides*.

The number of receptor RBCs induced by *C. ambrosioides* volatile stress showed a dual effect of time and concentration, with low concentrations of volatiles promoting RBC production and high concentrations inhibiting RBC production (Figure 4). The growth rate of the RBC number slowed down with treatment time and was finally even lower than that of the control. Thus, the promoting effect of volatile oil and α-terpinene from *C. ambrosioides* on the number of receptor RBCs was transient. Relative to the control treatment, the highest rate of increase in the number of RBCs was 2.61 and 2.74 times higher in *Z. mays* and *G. max*, respectively, 2.95 and 2.77 times higher in the volatile oil-treated *Z. mays* and *G. max* groups, respectively, and 3.20 and 3.31 times higher in the α-terpinene-treated *Z*. *mays* and *G*. *max* groups, respectively. The chemosensory potential of the α-terpinene treatment group was 1.08 and 1.19 times higher than that of the essential oil treatment group for *Z. mays* and *G. max*, respectively.

### 3.5. Differentially Expressed Genes Associated with RBC Release

The transcriptome results using the Illumina HiSeq platform were highly reliable (Figure S1). In this study, removal and non-removal of RBCs from plants exposed to volatiles from *C. ambrosioides* revealed DEGs associated with the release of RBCs in the root tips of two receptor plant species (i.e., from comparisons ZCKN-vs-ZCKW, ZVN-vs-ZCKN, ZTN-vs-ZCKN; GCKN-vs-GCKW, GVN-vs-GCKN, GTN-vs-GCKN). The expression of DEGs in the root tips of the two receptor plants after the removal of RBCs was generally up-regulated, but such changes were almost entirely statistically non-significant, indicating that the root tips of the receptor plants can replenish RBCs after the removal of RBCs. However, the changes in the expression of DEGs related to the release of RBCs were significantly higher after treatment with *C. ambrosioides* volatiles compared to the ZCKN-vs-ZCKW and GCKN-vs-GCKW comparisons, indicating that the release of RBCs from the root tip of the receptor plant was accelerated under the stress induced by *C. ambrosioides* volatiles. Therefore, in a follow-up assay, 11,847 up-regulated and 7051 down-regulated DEGs were identified in two comparisons of *Z*. *mays* groups (ZVN-vs-ZCKN and ZTN-vs-ZCKN). In the *G. max* treatment groups (GVN-vs-GCKN, GTN-vs-GCKN), a total of 3637 up-regulated and 3427 down-regulated DEGs were identified. GO enrichment analysis and KEGG pathway analysis were performed for the differentially expressed genes (Figures S2–S9). The top 10 significantly enriched GO terms in the biological process (BP), molecular function (MF), and cellular component (CC) classifications revealed that significantly up-regulated DEGs in *Z. mays* were mainly related to the oxidation-reduction process, catabolic processes (BP), oxidoreductase activity, DNA-binding transcription factor activity (MF), plasma membrane, and cell periphery (CC), and down-regulated DEGs were significantly enriched in translation, xyloglucan metabolic process (BP), cell wall, extracellular region and non-membrane bound organelles (CC), structural molecular activity, and ribosomal structural component (MF) in *Z. mays*. Similarly, significantly up-regulated DEGs in *G. max* were mainly involved in glutathione metabolism, sulfur compound metabolism (BP), plasma membrane, photosystem II (CC), and glutathione transferase activity (MF), and down-regulated DEGs were significantly enriched in redox process, nucleosome assembly (BP), extracellular region and nucleosome (CC), nucleosome DNA binding, and oxidoreductase activity (MF). GO analysis showed that volatiles from *C. ambrosioides* effectively enhanced aspects of the oxidation-reduction process, glutathione metabolism, ethylene activation pathways, composition and regulation of the plasma membrane and cell periphery, as well as chloroplast and photosynthetic systems in the root tips of both *Z*. *mays* and *G*. *max*, thereby improving the receptors’ tolerance to volatiles. To further determine the biological pathways induced by volatile stress, all DEGs were subjected to KEGG pathway enrichment analysis. In the comparison group of two receptors, more DEGs were significantly enriched in metabolic pathways and biosynthesis of secondary metabolites. KEGG analysis showed that when exposed to volatiles from *C. ambrosioides*, receptor amino acid metabolism, carbohydrate metabolism, and other processes were up-regulated to reduce the toxicity of volatiles to the receptor plants.

**Figure 5:**
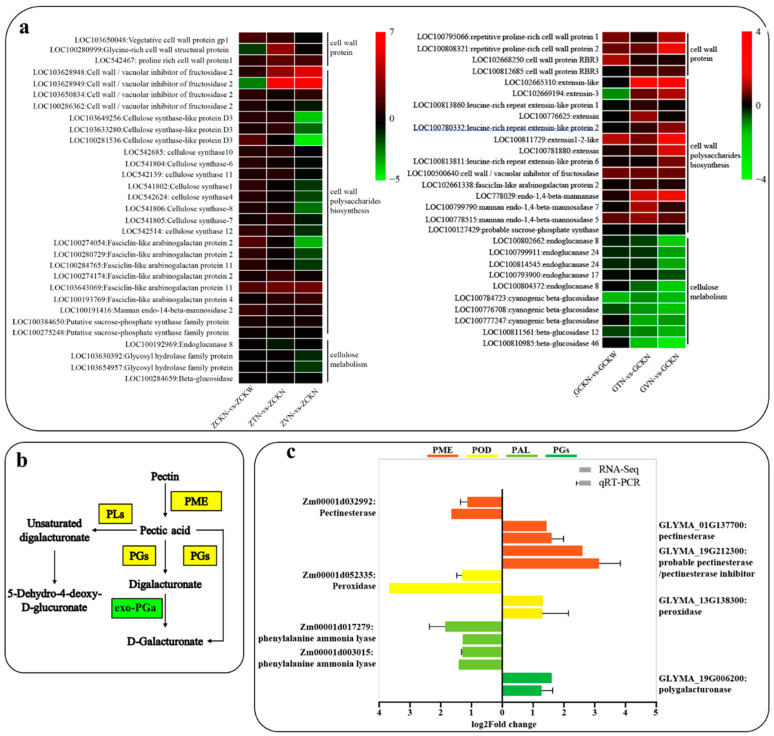
(**a**) The log_2_ (fold change) in the expression of differentially expressed genes (DEGs) (red shows up-regulated DEGS, and green shows downregulated DEGs) related to RBC wall metabolisms between the sample pairs under the stress of volatile oil and α-terpinene from *Chenopodium ambrosioides*. (**b**) Schematic diagram showing pectin degradation by pentose and glucuronate interconversions. The green box indicates the downregulated DEGs, while the yellow box indicates the up-regulated and downregulated DEGs. (**c**) Validation of RNA-Seq data by RT-qPCR. Eight representative genes were chosen to validate the RNA-Seq data by RT-qPCR. The bars with whiskers represent mean and standard deviation values of the log_2_ (fold change) obtained from three biological replicates of RT-qPCR. The bars without whiskers show the corresponding data based on RNA-Seq. Note: PME, pectin methylesterases; POD, peroxidase; PAL, phenylalanine ammonia-lyase; PGs polygalacturonase.

Root cap PME plays a crucial role in the release of RBCs. Significantly enriched pectin degradation pathways were found in two comparisons of groups of the two receptor plants. 9 (6 up-regulated and 3 down-regulated) and 7 (3 up-regulated and 4 down-regulated) DEGs belonged to the pectin degradation pathway in *Z. mays* (ZVN-vs-ZCKN, ZTN-vs-ZCKN), whereas in *G. max* (GVN-vs-GCKN, GTN-vs-GCKN), 15 (7 up-regulated and 8 down-regulated) and 6 (3 up-regulated and 3 down-regulated) DEGs belonged to this pathway, including PMEs, pectin lyases (PLs), and endo-polygalacturonases (PGs), respectively. The PME activity assay revealed that PME activity was increased after volatile matter stress by *C. ambrosioides* (Figure 3). In addition to the pectin degradation pathway, we found that many DEGs are involved in cell wall component remodeling. In the *Z. mays* (ZVN-vs-ZCKN, ZTN-vs-ZCKN) and *G. max* (GVN-vs-GCKN, GTN-vs-GCKN) comparison groups, 31 and 28 DEGs involved in the metabolism of cell wall construction were detected (Figure 5a), and these DEGs encode a number of enzymes, mainly including cellulose synthase (CesA), endoglucanases (EGs), and beta-glucosidases (BGLs), among others.

The above results indicate that the synthesis of the plant receptor cell wall material is up-regulated and that the activity of PME is increased under volatile stress induced by *C. ambrosioides*, which reduces intercellular adhesion and thus promotes the release of RBCs.

### 3.6. Effect of C. ambrosioides Volatiles on Cellular Activity and Intracellular ROS Levels in RBCs of Receptor Plant Roots

**Figure 6:**
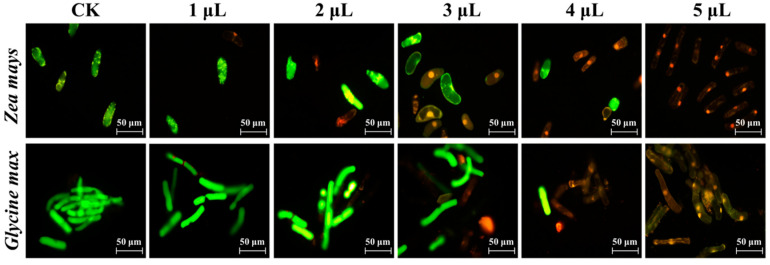
Fluorescence micrograph of the activity of receptor root border cells under the stress of volatiles from *Chenopodium ambrosioides*. The concentrations of the treatments are indicated above each column of images in the figure.

After the two kinds of receptor RBCs were treated with different concentrations of volatiles and subjected to AO/EB staining, the cells in the control group showed green fluorescence with clear cell outlines (indicating cell survival). In contrast, the cells in the treatment group showed varying degrees of light orange-red fluorescence, with the nuclei showing bright orange-red fluorescent spots, and some of the nuclei were lysed (Figure 6), indicating the loss of activity of RBCs and the characteristics of apoptosis. With the increase in volatile oil and α-terpene concentration, the death rate of *Z. mays* and *G. max* RBCs increased significantly. When the volatile substance concentration was the highest, the death rate of *Z. mays* and *G. max* RBCs reached 70.90% and 71.43% in the volatile oil treatment group and 60.79% and 58.08% in the α-terpene treatment group, respectively. The toxic effect of α-terpene treatment on *Z. mays* and *G. max* RBCs was 0.86 and 0.81 times that of the volatile oil treatment group, respectively (Figure S10).

**Figure 7:**
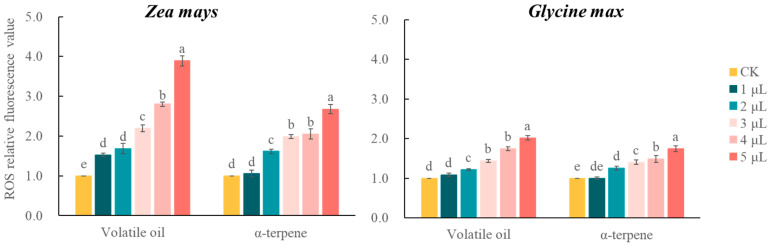
Effects of volatile oil and α-terpinene from *Chenopodium ambrosioides* on the relative content of reactive oxygen species in receptor plant root border cells.

The intracellular ROS content in RBCs increased continuously as the concentrations of the volatile treatments increased. When the concentration of volatile oil and α-terpinene reached the maximum, the relative fluorescence values of ROS in RBCs of both *Z. mays* and *G. max* reached the maximum, which were 3.89 and 2.02 times higher in the volatile oil-treated group than the control group, respectively, and 2.67 and 1.74 times higher in the α-terpinene-treated group than the control group, respectively. At this same time point, the RBC ROS contents of *Z. mays* and *G. max* in the α-terpinene-treated group were 0.69 and 0.86 times that of the volatile oil-treated group, respectively. The above results indicated that the volatiles of *C*. *ambrosioides* induced oxidative damage in the RBCs of the receptor plants (Figure 7).

### 3.7. Changes in Ultrastructure and Autophagy of Receptor RBCs under Treatment with C. ambrosioides Volatiles

#### 3.7.1. Changes in Ultrastructure

**Figure 8:**
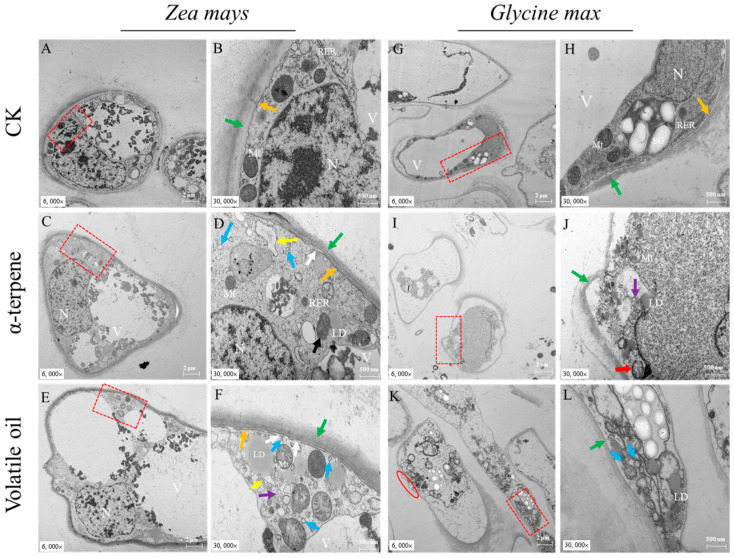
Transmission electron micrographs of intracellular autophagosomes in root border cells of two receptor plant species under the stress of volatile oil and α-terpinene from *Chenopodium ambrosioides*. Vesicles, blue arrows; autophagy, purple arrow; mitochondrial autophagy, black arrow; mitochondria swelling, red arrow; discontinuous cell membrane, white arrow; cell wall, green arrow; cell membrane, orange arrow; rough endoplasmic reticulum expanded, yellow arrow; no cell wall area, cell wall dissolution, and cell wall dissolution thinning, red oval area; Mitochondria, Mt; rough Endoplasmic Reticulum, RER; Nucleus, N; Lipid droplets, LD; Vacuole, V; Starch granules, SG. The enlarged image corresponding to the red box on the left is the image on the right. The mapping is as follows: (**A**) to (**B**; **C**-**D**; **E**-**F**; **G**-**H**; **I**-**J**; **K**-**L**).

In *Z. mays* RBCs, the control group had normal cell morphology, abundant organelles, obvious vesicles, continuous cell membranes, and intact and clear cell wall structures (Figure 8A,B). The α-terpinene-treated cells had polygonal nuclei, clear nuclear membranes, and more abundant organelles in their cytoplasm, and a small number of lipid droplets, autophagy (autophagosomes and mitochondrial autophagy), and rough endoplasmic reticulum could be observed (Figure 8C,D). In the volatile oil-treated group, some mitochondria were swollen (cristae were dissolved and broken), cell membranes were discontinuous, the outer layer of the cell wall was irregular, and the rough endoplasmic reticulum was slightly dilated (Figure 8E,F).

In *G. max* RBCs, the control group had normal cell morphology, clear nuclear membranes, cytoplasm containing organelles such as mitochondria, chloroplasts (with starch granules), rough endoplasmic reticulum, and ribosomes, and an intact cell wall structure (Figure 8G,H). In the α-terpinene stress group, the cell morphology was more abnormal in structure, with discontinuous nuclear membranes, a small amount of swollen mitochondria visible in the cytoplasm, a small amount of lipid droplets, and starch; additionally, the nuclear membrane was discontinuous, with some swollen mitochondria, some lipid droplets, and starch granules (presumably associated with the abnormal chloroplast structure, i.e., residual starch granules); autophagic vesicles were observable in the cytoplasm, the cell membrane was surrounded by secretory vesicles, and part of the cell membrane structure was destroyed (Figure 8I,J). In the volatile oil-treated group, the cell membrane was more continuous, the cell wall structure was dissolved, some cells had no cell wall in some areas, the vesicles were more obvious, lipid droplets and autophagic vesicles were visible, and there were a large number of secretory vesicles (Figure 8K,L).

The above results indicated that after the volatile oil and α-terpinene treatment, the secretion of autophagic vesicles and vesicles in the RBCs of the receptor plant roots increased significantly, and some cells showed abnormal morphological structures, discontinuous nuclear membranes, swollen mitochondria, and disrupted cell membrane structures; thus, the cells appeared in a necrotic state.

#### 3.7.2. Changes in Autophagy

**Figure 9:**
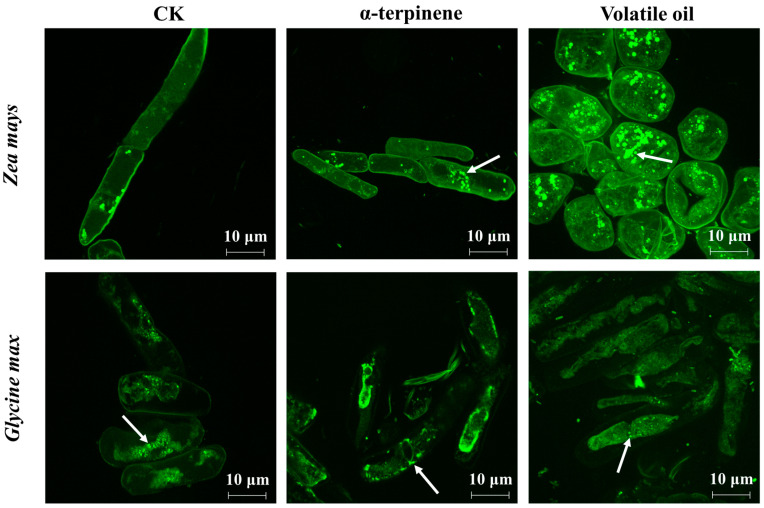
Autophagic vesicles of two receptor plant root border cells induced by volatile oil and α-terpinene from *Chenopodium ambrosioides.* Autophagic vesicles, white arrow.

Indications of autophagy could be observed by laser confocal microscopy in the RBCs of both receptor plants treated with *C. ambrosioides* volatiles (Figure 9), and bright green, fluorescent vesicles, i.e., autophagic vesicles appeared in the RBCs of both receptors. The number of autophagic vesicles was significantly higher in the volatile oil-treated group than in the control group, and the number of autophagic vesicles was highest in the volatile oil-treated group, followed by the α-terpinene-treated group.

**Figure 10:**
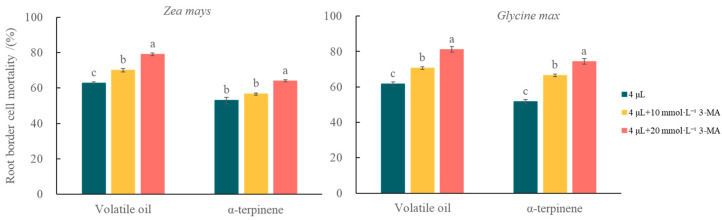
The effect of 3-methyladenine on the effect of volatile oil and α-terpinene from *Chenopodium ambrosioides* in inducing death of root border cells of receptor plants.

To further demonstrate the mitigating effect of autophagy on volatile stress, a mixture of autophagy inhibitor (3-MA) and volatile oil was used to treat the RBCs of the two receptor plants (Figure 10). Compared with volatile oil treatment alone, RBC death was significantly (*p* < 0.05) increased in the co-treated groups of *Z. mays* and *G. max* and was positively correlated with the concentration of autophagy inhibitor; additionally, when the 3-MA concentration reached 20 mmol/L, the RBC mortality in *Z. mays* and *G. max* was 1.26 and 1.32 times higher in the combined volatile oil + autophagy inhibitor treatment group. The RBC mortality in the α-terpinene + autophagy inhibitor treatment group of *Z.mays* and *G. max* was 1.21 and 1.44 times higher than that in the α-terpinene treatment alone, respectively. The mortality rate of the α-terpinene + autophagy inhibitor-treated groups was 0.96 and 1.09 times higher than that of volatile oil + autophagy inhibitor-treated groups in *Z. mays* and *G. max* roots, respectively.

The volatile chemosensory stress induced by *C. ambrosioides* volatiles activated the autophagic process of RBCs of receptor plants, and autophagy had a mitigating effect on RBC death.

## 4. Discussion

### 4.1. Volatile Allelopathic Stress from C. ambrosioides Alters the Production and Release of Receptor Plant RBCs

As an important protective structure of plant root systems, especially the root tip, the RBCs and their extracellular mucilage layer can chelate external stressors and thus protect the root tip [47]. In this study, both receptor plants showed significantly shorter root lengths and slower growth under treatment with *C. ambrosioides* volatiles. Similar phenomena have been repeatedly found in many plants under allelochemical stress [48], suggesting that the volatiles of *C. ambrosioides* showed similarly strong effects. At the same time, we observed that the area of root extracellular trap in the root cells of two recipient plants significantly increased compared to the control group under the stress of volatile oil and α-terpinene from *C. ambrosioides*. The expansion of root extracellular traps in the root cells is considered to play a crucial role in plant root tip defense, and the extracellular DNA contains antimicrobial compounds that can block the invasion of pathogens in the external environment while chelating to alleviate the stress effect of exogenous substances [49,50]. When inhibited by DNA enzymes, the root extracellular trap structure is broken, allowing bacteria and allelochemicals to break through this defense barrier and directly cause damage to the root tip [51]. Therefore, in our research, it was found that, when we removed the RBCs (N group), the root lengths of receptor plants were significantly shorter than the treated group in which we retained the RBCs (W group), especially in the medium concentration treatment groups (V2 and T2). The above results indicated that the production and formation of RBCs had a protective effect on the root system of the receptor plants under allelopathic stress from *C. ambrosioides*. As a “defense cell,” RBCs have been confirmed to exhibit accelerated production and release in response to low-intensity external stress [22,26]. In *Pisum sativum*, fucosylation of xyloglucan (XyG) is required for proper formation of RBCs morphology and its release [52]. The *Arabidopsis* NIN-LIKE PROTEIN 7 (NLP7) transcription factor (NAC) is involved in the maturation and separation process of RBCs. This transcription factor regulates cell wall composition by controlling the expression of several cell wall modifying enzymes, including the cellulase CEL5, and NAC-deficient or -deletion mutants that exhibit retention of the cells in the last layer of the root cap [53]. These results suggested that cell wall formation and cell wall polysaccharide metabolism were closely related to the proper production and release of RBCs. In this study, 24 and 14 DEGs from *Z. mays* and *G. max*, respectively, were identified to be involved in cell wall polysaccharide metabolism. Among them, lysine-rich extensin forms a positively charged scaffold in the cell plate that can provide an ordered deposition template for new cross walls during cytoplasmic division [54]. Mannan (endo-1,4-β-mannosidase) is a key component of lignocellulose present in the hemicellulose fraction of the primary plant cell wall [55], and its production is associated with cell wall synthesis. Arabinogalactan proteins play an important role in cell wall formation [56]. In summary, under allelopathic stress induced by *C. ambrosioides* volatiles, receptor plants can build cell walls and accelerate the formation of RBCs by accelerating the production of proteins such as FLAs and extensin and increasing the activity of enzymes such as cellulase.

The intercellular layer of plants is rich in pectic polysaccharides, particularly the pectic polysaccharide high galacturonic acid (HG) [57], and blocking the synthesis of HG alters the organization of RBCs in *Arabidopsis* [58]. The isolation of *P. sativum* and *Arabidopsis* RBCs requires post-synthetic demethylesterification of HG by pectin methylesterase (PME) [59]. The present study observed a significant increase in DEGs in the pectin degradation pathway, with a total of 11 DEGs associated with the pectin degradation pathway in the *Z. mays* treatment group, in which both key genes encoding PME and PGs that degrade HG were activated. Similarly, in the *G. max* treatment group, a total of 18 DEGs were associated with the pectin degradation pathway, in which key genes encoding PME and PGs were also both activated. The changes in PME activity revealed by the comparative transcriptome analyses were supported by our experimental validation (Figure 3). It was shown that the root tips of the two receptor plants under *C. ambrosioides* volatile stress altered gene expression patterns, increased the activity of enzymes such as PME, accelerated the degradation of adhesion components (such as HG and pectin in the intercellular layer), and promoted the release of RBCs. Based on the above experimental results, we speculate that *Z. mays* and *G. max* root tips resisted *C. ambrosioides* volatile stress by accelerating the production and release of RBCs, but this trend of increasing RBCs decreased as treatment time continued, indicating that the resistance mechanism of the receptors was sensitive and rapid. Compared with *G. max*, there was a lag in the trend of the change in root length and RBC number in *Z. mays*, and this phenomenon was also evident in the DEGs of the proteins involved in cell wall formation and cell wall polysaccharide metabolism. The DEGs involved in this process were much more up-regulated in *G. max* than in *Z. mays*, and, in the pectin degradation pathway, the expression level of key enzyme genes involved in pectin degradation was higher in *G. max* than in *Z. mays.* This pattern may be owing to the better tolerance of *Z. mays*, as an introduced exotic species, compared to *G. max* (native species) to the allelopathic stress of volatiles from *C. ambrosioides*.

The composition of *C. ambrosioides* volatile oil is complex, and α-terpinene is one of its most abundant components; moreover, our previous study confirmed that this substance is the main allelochemical in *C. ambrosioides* [36]. The present study showed that the effect of α-terpinene on root length and ROS content of RBCs was about 70% of the effect of volatile oil, and the effect on root cap PME activity and RBC number and activity was greater than that observed in the volatile oil treatment group. These results further confirmed the previous findings, indicating that α-terpinene plays an important role in the allelopathy of *C. ambrosioides* among its various volatile components.

### 4.2. Autophagy Occurs in RBCs under Volatile Stress from C. ambrosioides

Allelochemicals induce a burst of ROS in receptor cells, and the resulting oxidative damage is key to their toxicity [60]. A complex intracellular antioxidant system scavenges excess ROS and maintains dynamic intracellular redox balance [61]. For example, extracts of *Mentha × piperita* L. increased the activity of peroxidase (POD) and other enzymes in *Raphanus sativus* L. seedlings, which in turn increased the ability of the seedlings to resist peppermint allelopathic toxicity [62]. However, the ability of cells to scavenge ROS is limited, and when cells are unable to resist ROS, it leads to ROS accumulation and oxidation of intracellular proteins, esters, and DNA, ultimately causing oxidative damage or even cell death [63]. In this study, the intracellular ROS content of two receptor plant species RBCs subjected to allelopathic stress induced by volatiles from *C. ambrosioides* was significantly increased; specifically, cellular activity was reduced, a large number of autophagic vesicles appeared intracellularly, various organelles exhibited different degrees of swelling and damage, and the volatile oil-treated group even showed discontinuity of nuclear and cellular membranes. Moreover, *G. max* RBCs also showed cell necrosis characteristics such as dissolution of its cell wall structure, which was consistent with the lower tolerance to physiological changes such as the changes in root length and the RBC number exhibited by *G. max* as a native species. Autophagy is involved in processes such as the response of plant cells to biotic and abiotic stresses [64] and the removal of damaged organelles, biomolecules, etc. by autophagy, which in turn promotes cell survival [65,66]. For example, under the influence of the ROS inducer methyl viologen, the content of oxidized proteins was increased and accumulated in large amounts in wild-type *Arabidopsis thaliana* plants, and autophagic activity was activated as indicated by a significant increase in the number of autophagic vesicles [67]. In the present study, we found that the number of autophagic vesicles in receptor RBCs significantly increased under *C. ambrosioides* volatile oil stress, while the mortality rate of RBCs increased in the volatile + autophagy inhibitor co-treatment group, clearly indicating that when exposed to *C. ambrosioides* allelopathic stress, RBCs scavenged harmful substances and damaged organelles produced by oxidative damage through autophagy, demonstrating that autophagy played an important role in defense against *C. ambrosioides* allelopathic stress.

## 5. Conclusions

Both *Z. mays* and *G. max* roots were significantly allelopathically inhibited by *C. ambrosioides* volatiles. Among these volatiles, α-terpinene played an important role in allelopathic stress, and the native crop species *G. max* was less tolerant to *C. ambrosioides* allelopathic stress than the introduced crop species *Z. mays*.

The presence of RBCs somewhat alleviated allelopathic stress from *C. ambrosioides*. When exposed to allelopathic stress from *C. ambrosioides*, the expression of genes related to cell wall substance synthesis and pectin degradation pathways was up-regulated, and PME activity was increased in RBCs of *Z. mays* and *G. max*, which accelerated the production and release of RBCs and increased the number of RBCs available to resist the allelopathic stress from *C. ambrosioides*.

RBC autophagy maintains cell survival to alleviate oxidative damage induced by allelopathic stress from *C. ambrosioides*. The number of autophagic vesicles was increased, and vesicle transport was accelerated in *Z. mays* and *G. max* RBCs under the influence of *C. ambrosioides* volatiles; accordingly, the cells cleared damaged materials and organelles by autophagy. However, when the intensity of allelopathic stress exceeded the tolerance limit of the receptor plants, the ROS content of receptor plant RBCs continued to increase, cell structure damage intensified, cell activity decreased, and cell mortality increased, which weakened the defense function of RBCs and eventually manifested as root tip growth retardation.

## Figures and Tables

**Table 1:** Experimental Design of Volatile Stress of *Chenopodium ambrosioides*.

Receptor Plants	Treatment			Comparisons
	Control	Volatile Oil	α-Terpinene	
*Zea mays*	ZCKN ZCKW	ZVN	ZTN	ZCKN-vs-ZCKW
				ZCKN-vs-ZTN
				ZCKN-vs-ZVN
*Glycine max*	GCKN GCKW	GVN	GTN	GCKN-vs-GCKW
				GCKN-vs-GTN
				GCKN-vs-GVN

Note: N, removal of root border cells (RBCs); W, presence of RBCs.

## Data Availability

The datasets generated during and/or analyzed during the current study are available from the corresponding author on reasonable request. These sequence data have been submitted to the GenBank databases under accession number PRJNA993366.

## Supplementary Materials

The following supporting information can be downloaded at: https://www.mdpi.com/article/10.3390/plants12223810/s1, Table S1 Primers and Sequences for qRT-PCR; Figure S1: Gene expression patterns of volatile matter treatment groups of two species compared with the control group (a) correlation analysis between different treatment groups of two species (b) principal component analysis (PCA) (c) Wien diagram comparing up-regulated and down-regulated DEG numbers (d) changes in total DEG numbers; Figure S2: Histogram for GO classification (a, b) annotation of the DEGs in the treatments ZVN-vs-ZCKN. The bubbles diagram (c) showed top 20 pathway of significantly enrichment of the up-regulated and down-regulated DEGs on GO; Figure S3: Histogram for KEGG pathway (a, b) annotation of the DEGs in the treatments ZVN-vs-ZCKN. The bubbles diagram (c, d) showed the top 20 pathways of significant enrichment of the up-regulated and down-regulated DEGs on KEGG; Figure S4: Histogram for GO classification (a, b) annotation of the DEGs in the treatments ZTN VS ZCKN. The bubbles diagram (c, d) showed top 20 pathway of significantly enrichment of the up-regulated and down-regulated DEGs on GO; Figure S5: Histogram for KEGG pathway (a, b) annotation of the DEGs in the treatments ZTN VS ZCKN. The bubbles diagram (c, d) showed the top 20 pathways of significant enrichment of the up-regulated and down-regulated DEGs on KEGG; Figure S6: Histogram for GO classification (a, b) annotation of the DEGs in the treatments GVN VS GCKN. The bubbles diagram (c, d) showed top 20 pathway of significantly enrichment of the up-regulated and down-regulated DEGs on GO; Figure S7: Histogram for KEGG pathway (a, b) annotation of the DEGs in the treatments GVN VS GCKN. The bubbles diagram (c, d) showed the top 20 pathways of significant enrichment of the up-regulated and down-regulated DEGs on KEGG; Figure S8: Histogram for GO classification (a, b) annotation of the DEGs in the treatments GTN VS GCKN. The bubbles diagram (c, d) showed top 20 pathway of significantly enrichment of the up-regulated and down-regulated DEGs on GO; Figure S9: Histogram for KEGG pathway (a, b) annotation of the DEGs in the treatments GTN VS GCKN. The bubbles diagram (c, d) showed the top 20 pathways of significant enrichment of the up-regulated and down-regulated DEGs on KEGG; Figure S10: Pectin methylase(PME) activity; Figure S11: Changes in the activity of receptor root border cells under the action of volatiles from *C. ambrosioides*.
