# Supplementary material for: Receptor Plants Alleviated Allelopathic Stress from Invasive Chenopodium ambrosioides L. by Upregulating the Production and Autophagy of Their Root Border Cells
Source: Plants (Basel). 2023 Nov 9;12(22):3810. doi: 10.3390/plants12223810 (PMC10674979; doi:10.3390/plants12223810)
Supplement: Supplementary file 1 [file plants-12-03810-s001.zip › plants-2677561-supplementary.pdf]

Table S1. Primers and Sequences for qRT-PCR

**Gene ID/name**	**Forward primer (5′-3′)**	**Reverse primer (5′-3′)**
Zm00001d003015	CCCGGGGAGATGAAGTGAAG	CAAACACGGGCAATGGTGTT
Zm00001d017279	CTCGAGTGCCTCAAGGAGTG	TCGAAGTTGGTTACAGGGCG
Zm00001d052335	GGCTCCAACAACGGACAGAT	ACATCAACGCGACGATCTCA
Zm00001d032992	GTCTACGTCGCCAAGACCAA	ACGTACCACAGCCGAATGTT
GLYMA_01G137700	CCCTATCCAAATGCCGTACCA	TCCATGTAGTGCTGAAGCTAGA
GLYMA_19G212300	GTCTTGGTACCCCTCGAAGC	GATCGATGTTGCAGCTGTGG
GLYMA_19G006200	ACAAACTGGTTGCTCGGACA	GACACGTGACTATACCTGCCA
GLYMA_13G138300	ACGAAACGGCAGTGTTTCCA	GTGTCTAATATCACCCCAAGTG TTT
Action1	GATTCCTGGGATTGCCGAT	TCTGCTGCTGAAAAGTGCTGAG
β-Actin	GTATTGGACTCTGGTGATGG	AGCAGAGGTGGTGAACAT

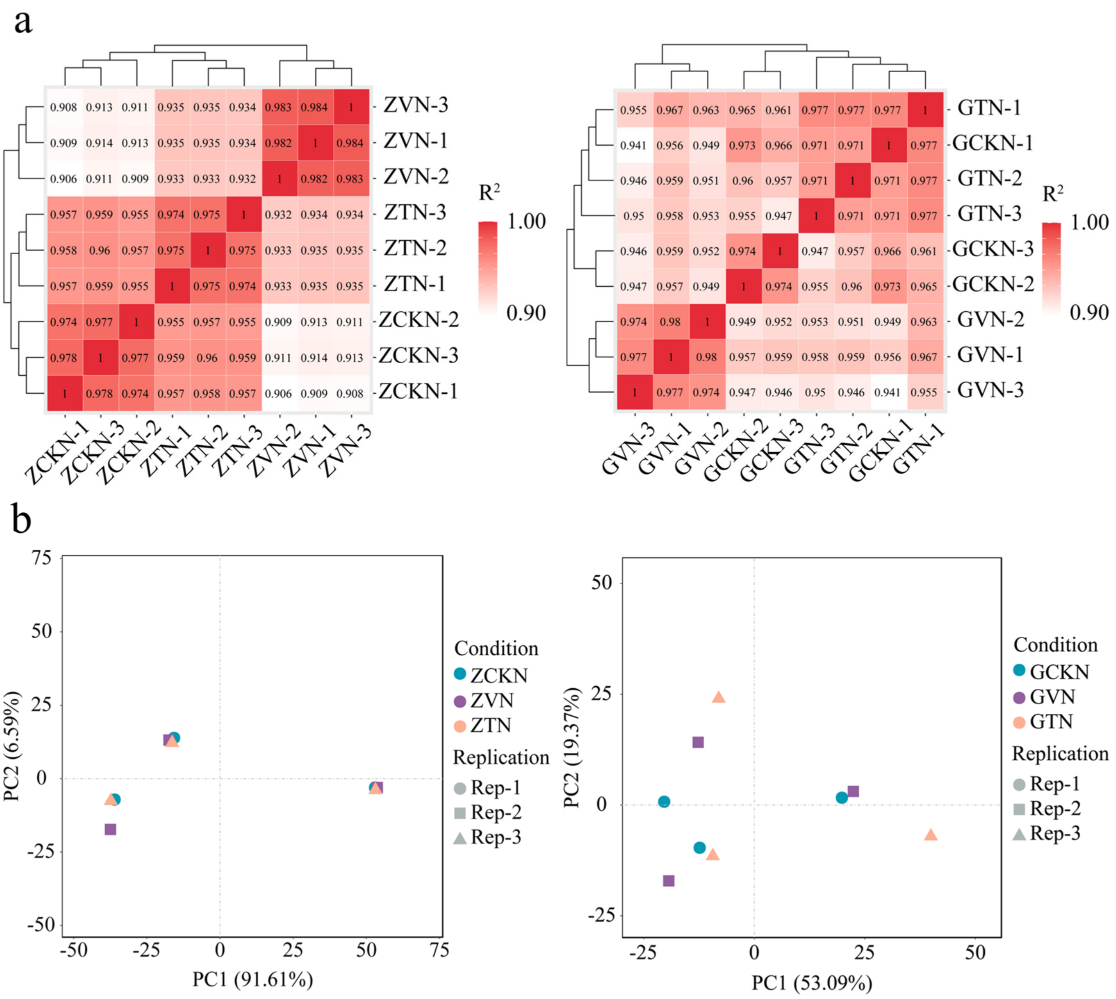

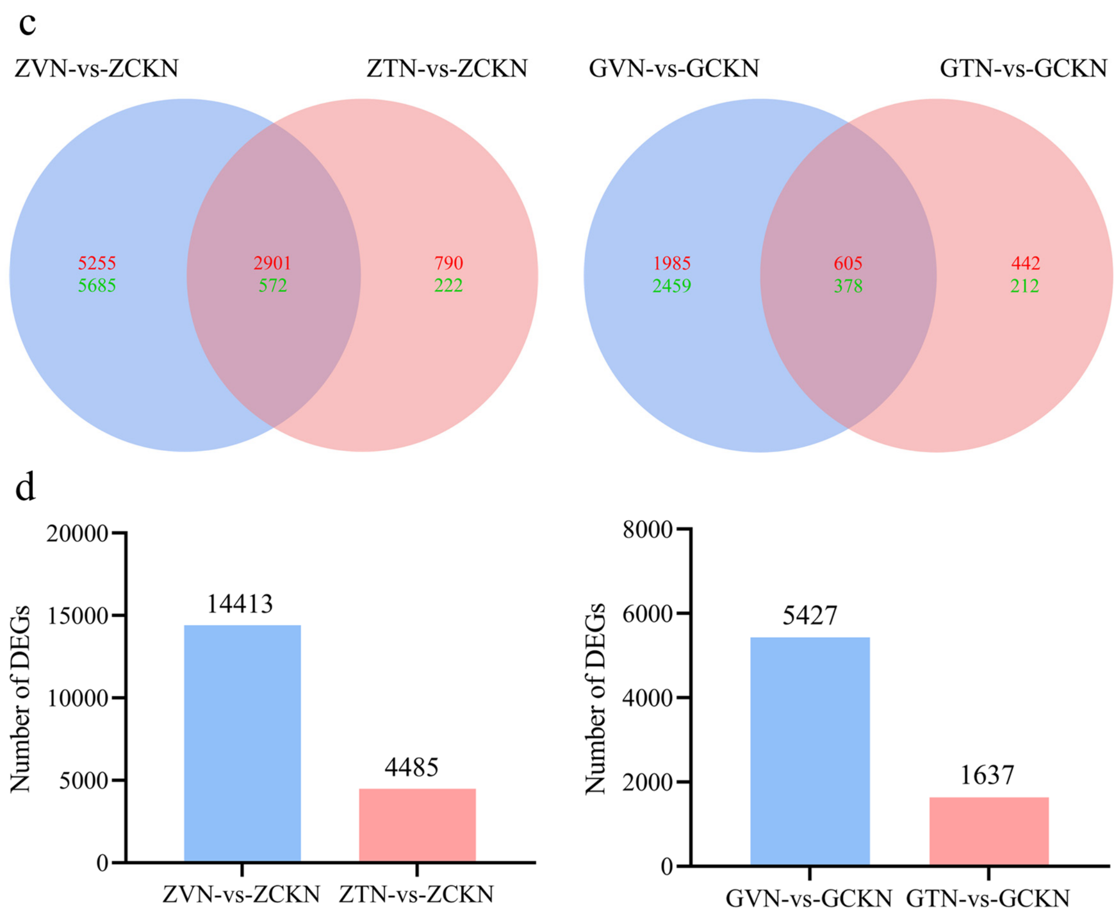

Figure S1. Gene expression patterns of volatile matter treatment groups of two species compared with the control group (a) correlation analysis between different treatment groups of two species (b) principal component analysis (PCA) (c) Wien diagram comparing up-regulated and down-regulated DEG numbers (d) changes in total DEG numbers

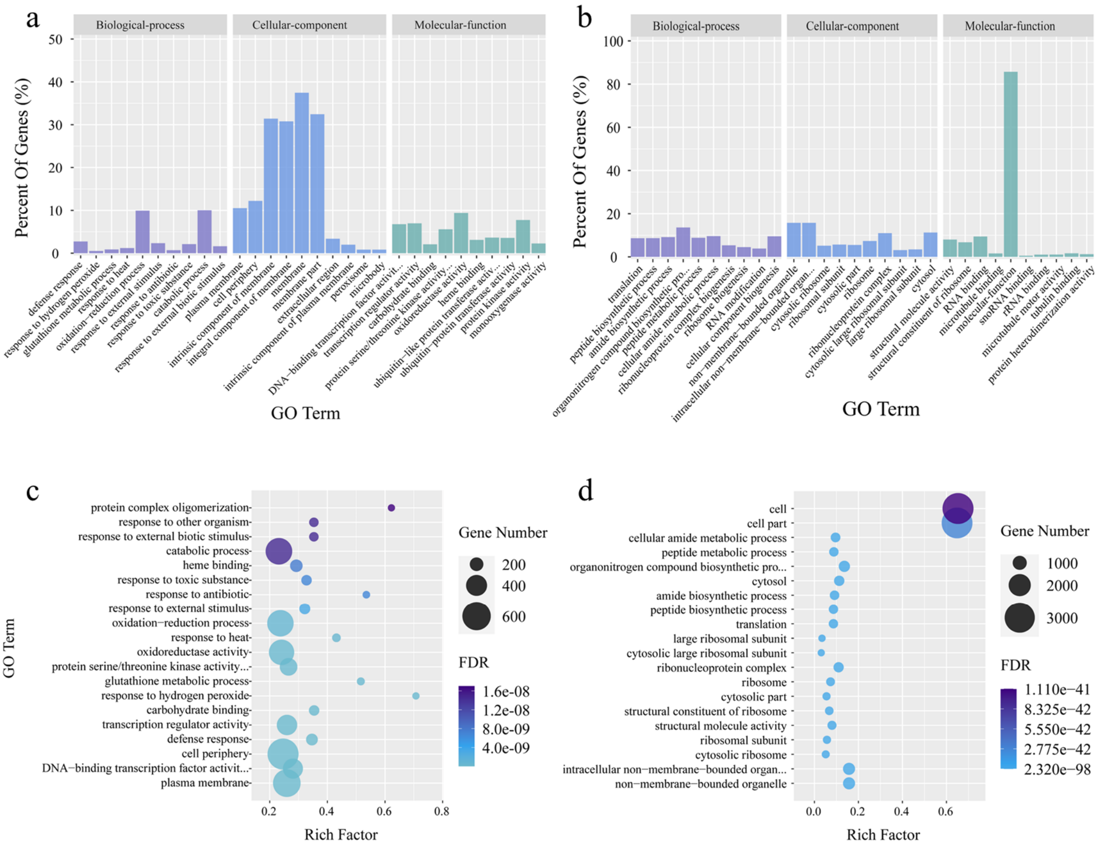

Up Down

Figure S2. Histogram for GO classification (a, b) annotation of the DEGs in the treatments ZVN-vs-ZCKN. The bubbles diagram (c) showed top 20 pathway of significantly enrichment of the up-regulated and down-regulated DEGs on GO.

Note: (a, b) The X-axis indicated the name of pathway, and the Y-axis represents the percent of genes involved DEGs in each pathway. (c,d) the X-axis was the rich factor, the Y-axis represents the name of the pathway. The bubbles size represents the number of DEGs involved. The bubbles color indicates the enrichment degree of pathway.

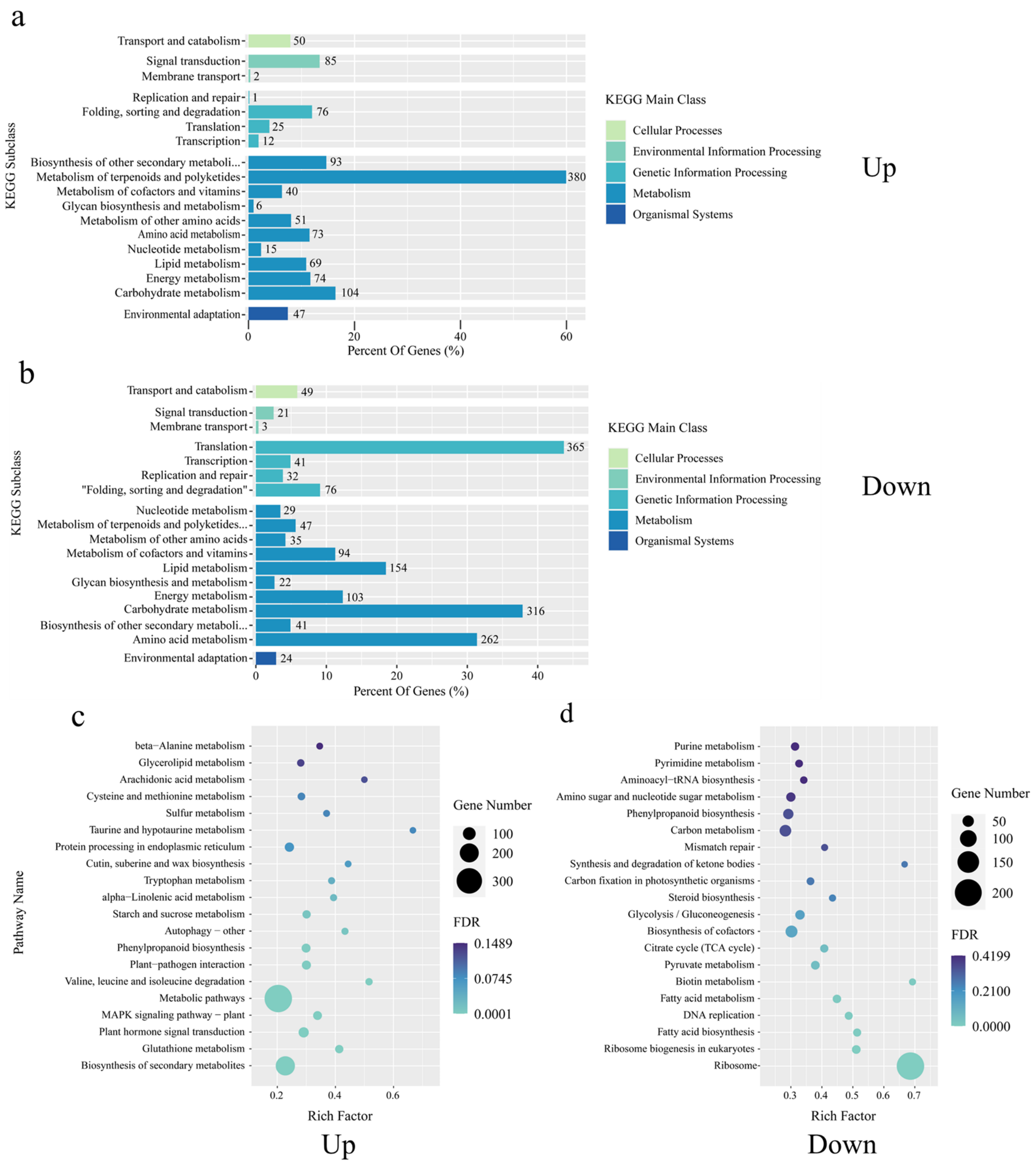

Figure S3. Histogram for KEGG pathway (a, b) annotation of the DEGs in the treatments ZVN-vs-ZCKN. The bubbles diagram (c, d) showed the top 20 pathways of significant enrichment of the up-regulated and down-regulated DEGs on KEGG.

Note: (a, b) The X-axis indicated the percent of genes involved DEGs in each pathway, and the Y-axis represents the name of the pathway. (c, d) the X-axis was the rich factor, the Y-axis represents the name of the pathway. The bubbles size represents the number of DEGs involved. The bubbles color indicates the enrichment degree of pathway.

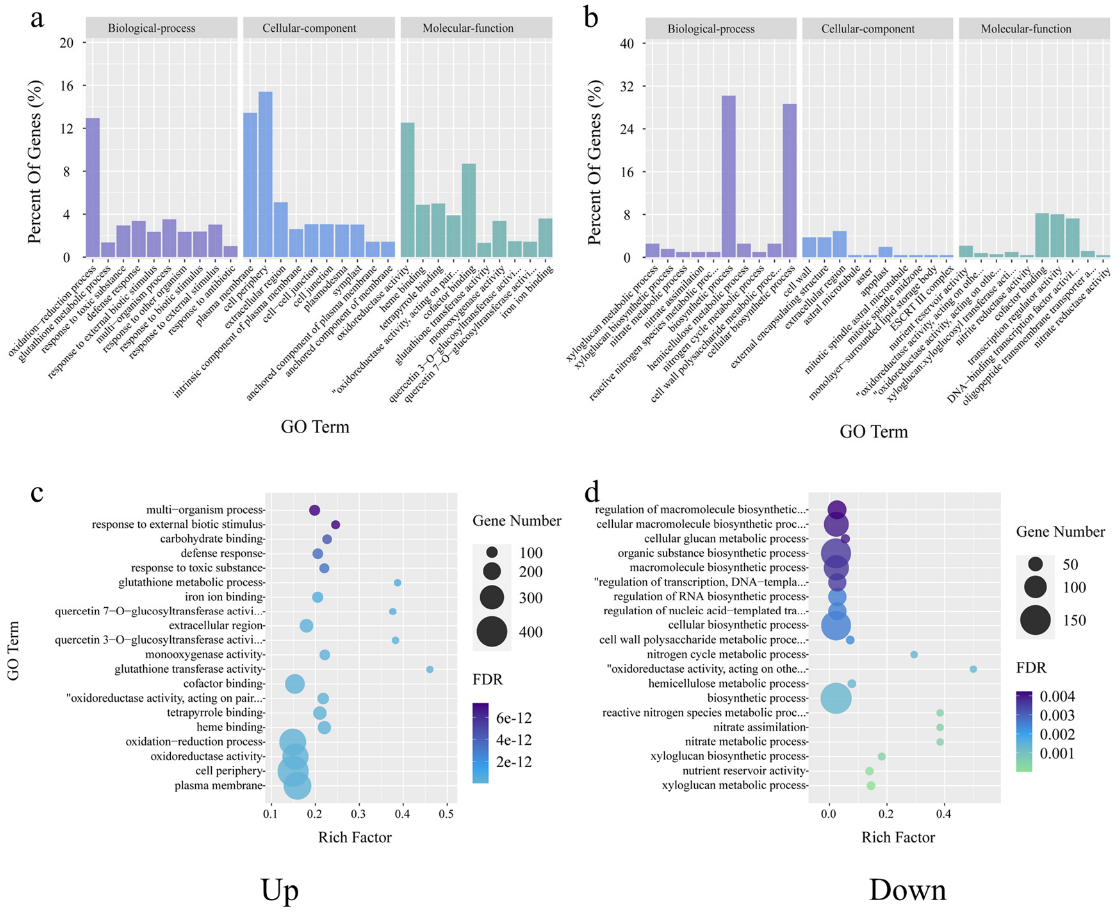

Figure S4. Histogram for GO classification (a, b) annotation of the DEGs in the treatments ZTN VS ZCKN. The bubbles diagram (c, d) showed top 20 pathway of significantly enrichment of the up-regulated and down-regulated DEGs on GO.

Note: (a, b) The X-axis indicated the name of pathway, and the Y-axis represents the percent of genes involved DEGs in each pathway. (c,d) the X-axis was the rich factor, the Y-axis represents the name of the pathway. The bubbles size represents the number of DEGs involved. The bubbles color indicates the enrichment degree of pathway.

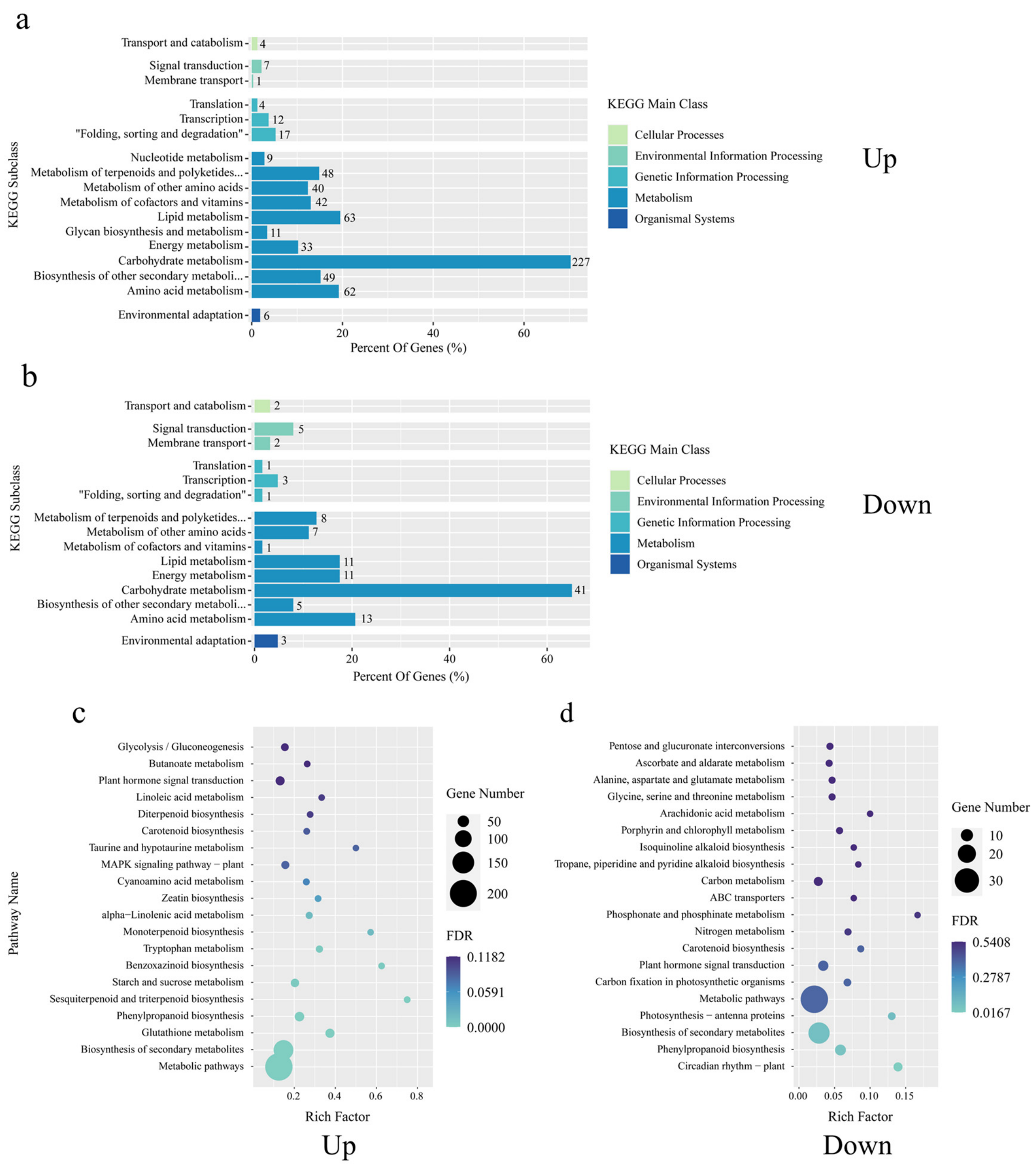

Figure S5. Histogram for KEGG pathway (a, b) annotation of the DEGs in the treatments ZTN VS ZCKN. The bubbles diagram (c, d) showed the top 20 pathways of significant enrichment of the up-regulated and down-regulated DEGs on KEGG.

Note: (a, b) The X-axis indicated the percent of genes involved DEGs in each pathway, and the Y-axis represents the name of the pathway. (c, d) the X-axis was the rich factor, the Y-axis represents the name of the pathway. The bubbles size represents the number of DEGs involved. The bubbles color indicates the enrichment degree of pathway.

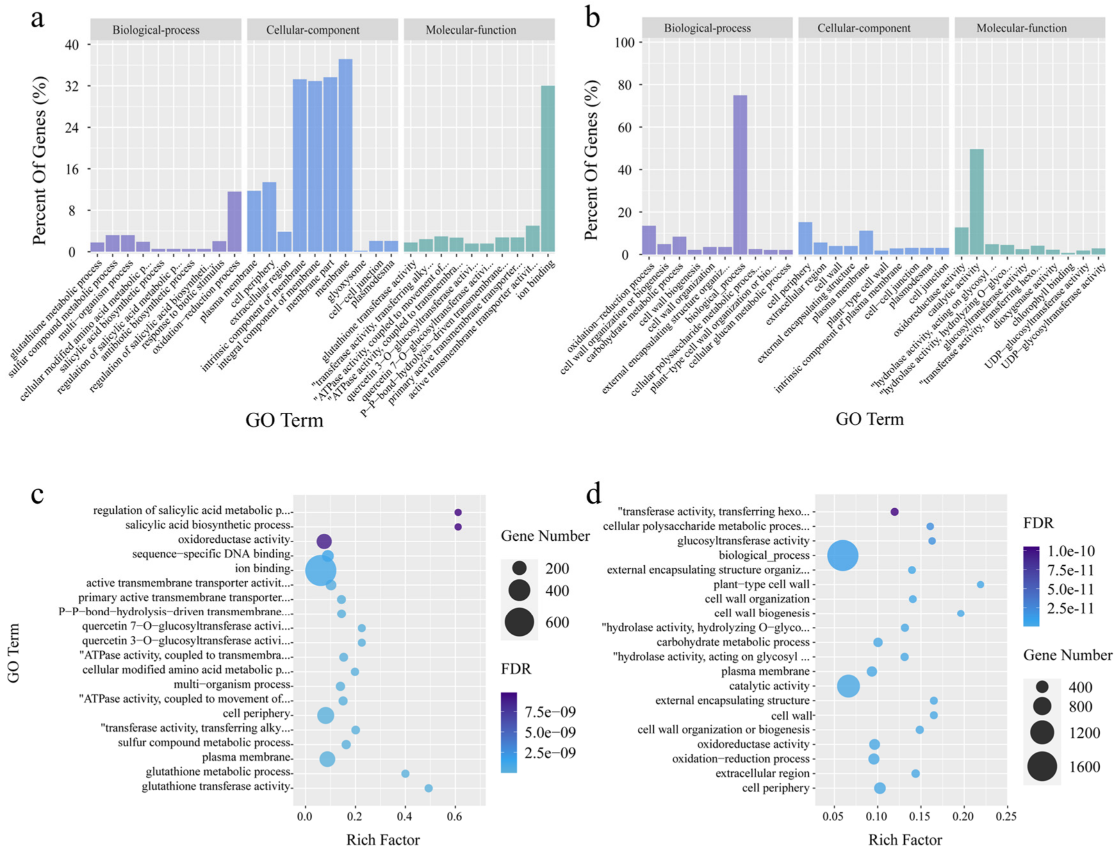

Up   Down

Figure S6. Histogram for GO classification (a, b) annotation of the DEGs in the treatments GVN VS GCKN. The bubbles diagram (c, d) showed top 20 pathway of significantly enrichment of the up-regulated and down-regulated DEGs on GO.

Note: (a, b) The X-axis indicated the name of pathway, and the Y-axis represents the percent of genes involved DEGs in each pathway. (c,d) the X-axis was the rich factor, the Y-axis represents the name of the pathway. The bubbles size represents the number of DEGs involved. The bubbles color indicates the enrichment degree of pathway.

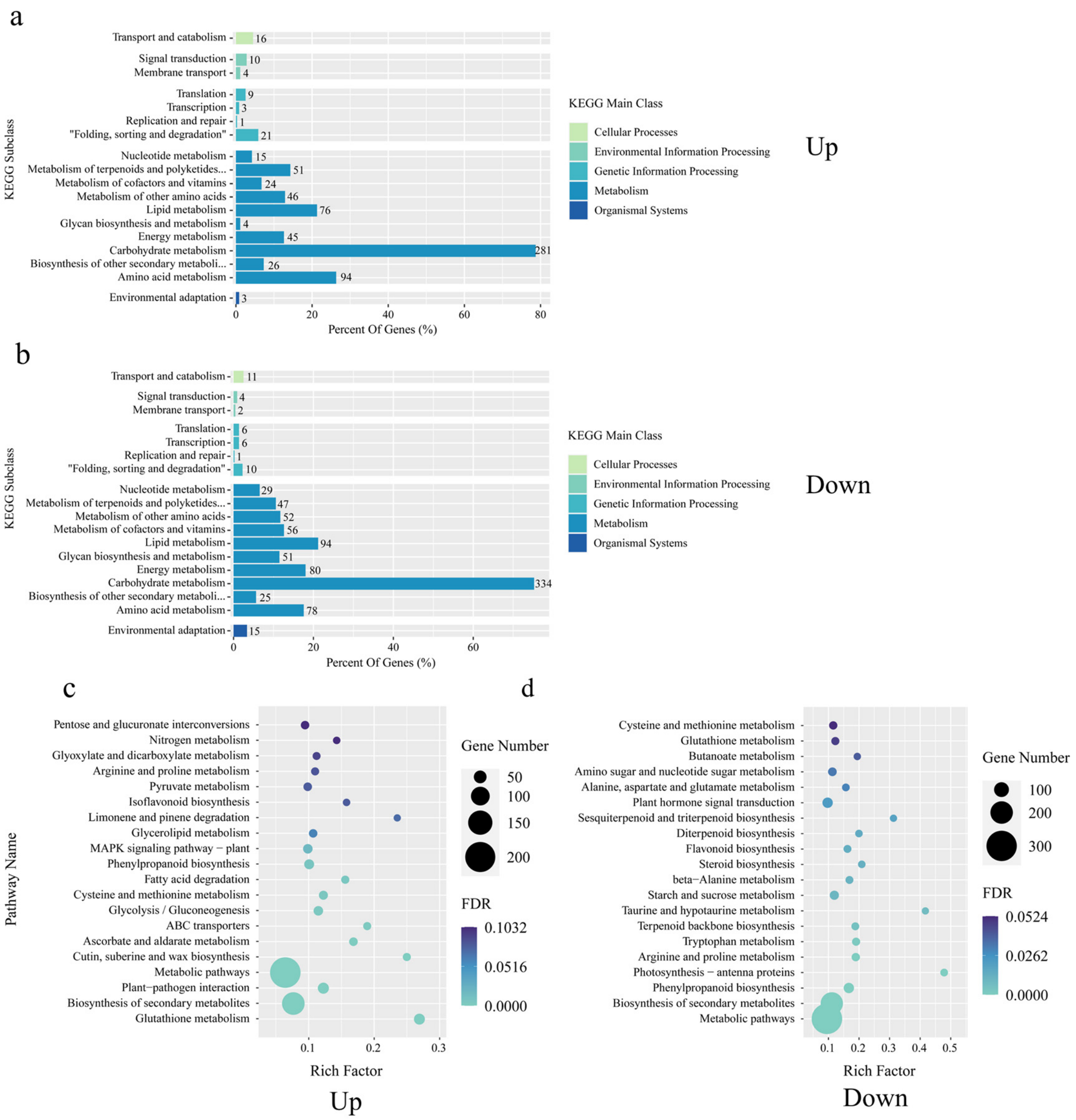

Figure S7. Histogram for KEGG pathway (a, b) annotation of the DEGs in the treatments GVN VS GCKN. The bubbles diagram (c, d) showed the top 20 pathways of significant enrichment of the up-regulated and down-regulated DEGs on KEGG.

Note: (a, b) The X-axis indicated the percent of genes involved DEGs in each pathway, and the Y-axis represents the name of the pathway. (c, d) the X-axis was the rich factor, the Y-axis represents the name of the pathway. The bubbles size represents the number of DEGs involved. The bubbles color indicates the enrichment degree of pathway.

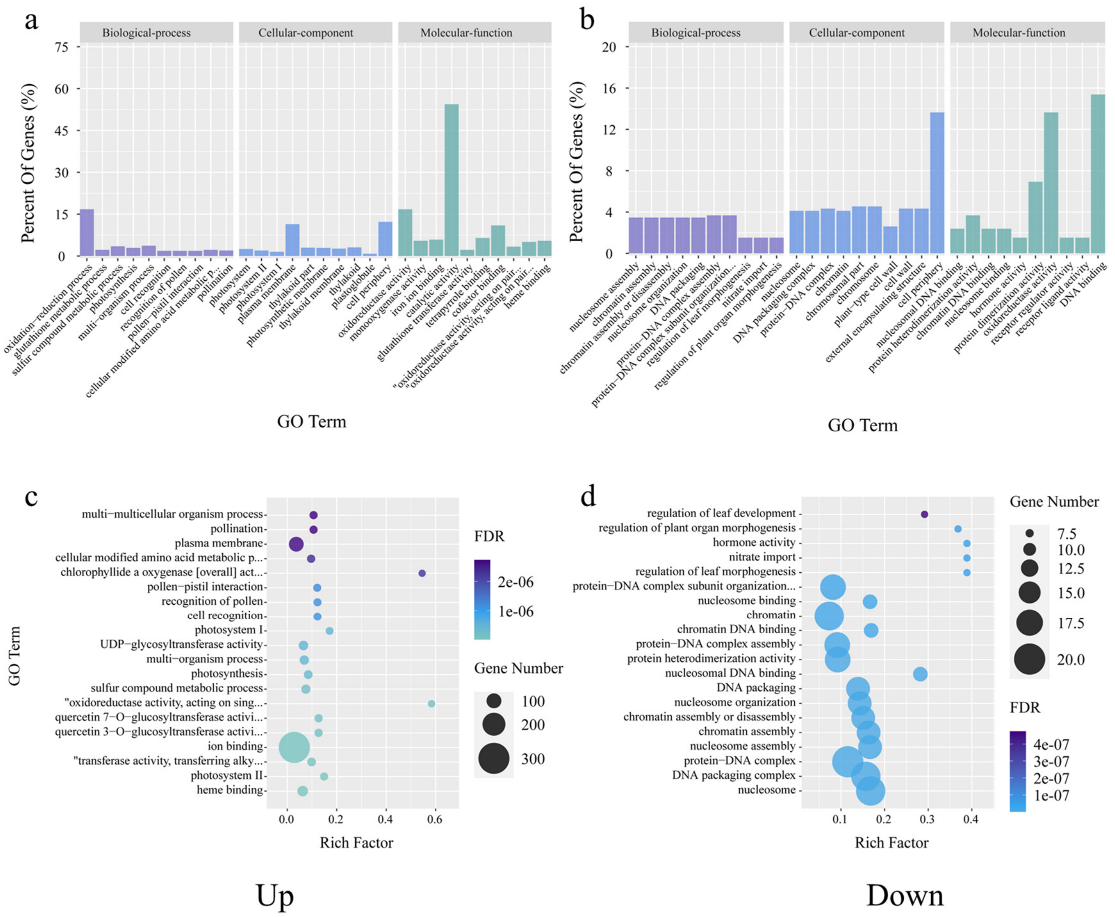

Figure S8. Histogram for GO classification (a, b) annotation of the DEGs in the treatments GTN VS GCKN. The bubbles diagram (c, d) showed top 20 pathway of significantly enrichment of the up-regulated and down-regulated DEGs on GO.

Note: (a, b) The X-axis indicated the name of pathway, and the Y-axis represents the percent of genes involved DEGs in each pathway. (c,d) the X-axis was the rich factor, the Y-axis represents the name of the pathway. The bubbles size represents the number of DEGs involved. The bubbles color indicates the enrichment degree of pathway.

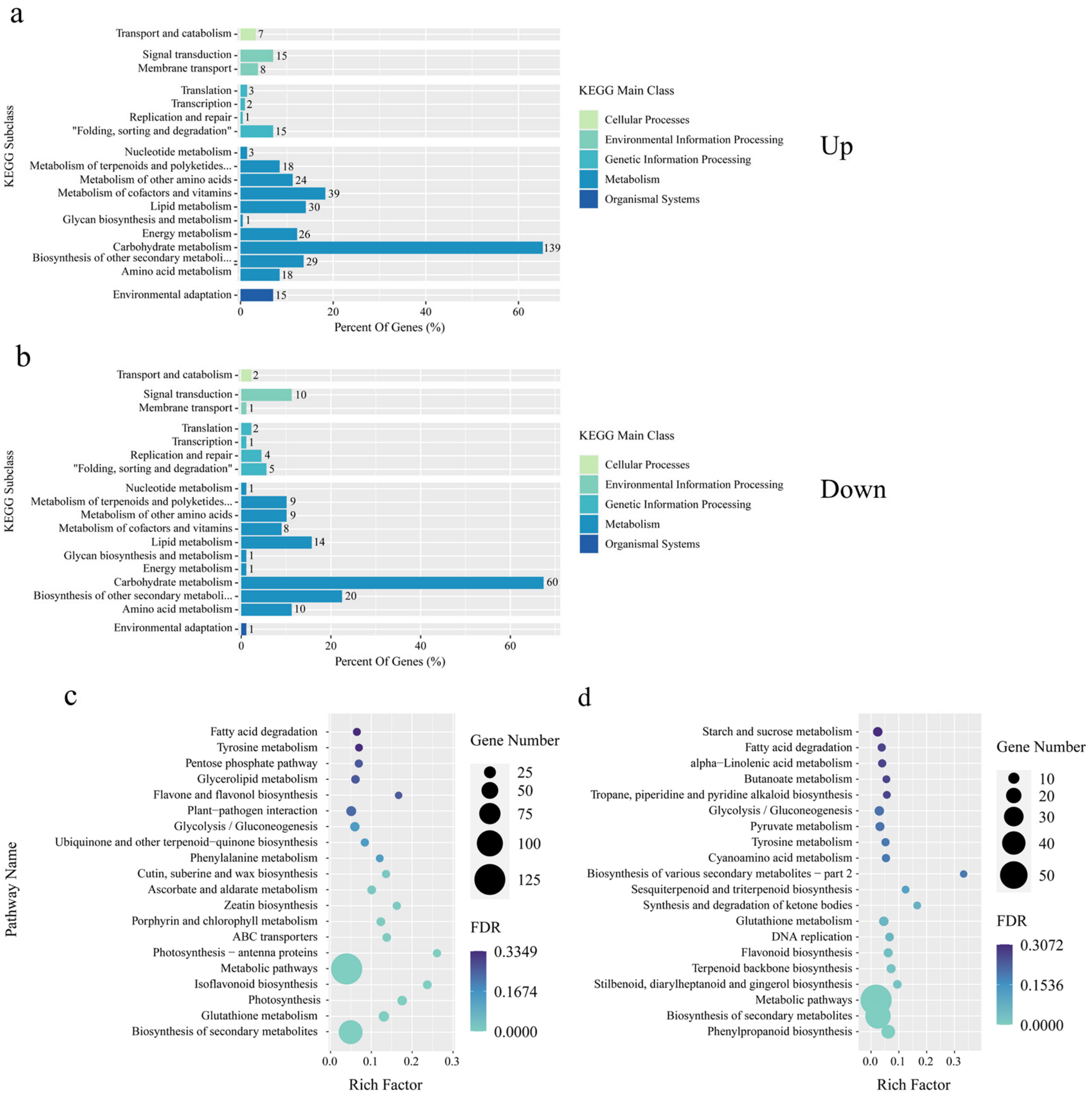

Figure S9. Histogram for KEGG pathway (a, b) annotation of the DEGs in the treatments GTN VS GCKN. The bubbles diagram (c, d) showed the top 20 pathways of significant enrichment of the up-regulated and down-regulated DEGs on KEGG.

Note: (a, b) The X-axis indicated the percent of genes involved DEGs in each pathway, and the Y-axis represents the name of the pathway. (c, d) the X-axis was the rich factor, the Y-axis represents the name of the pathway. The bubbles size represents the number of DEGs involved. The bubbles color indicates the enrichment degree of pathway.

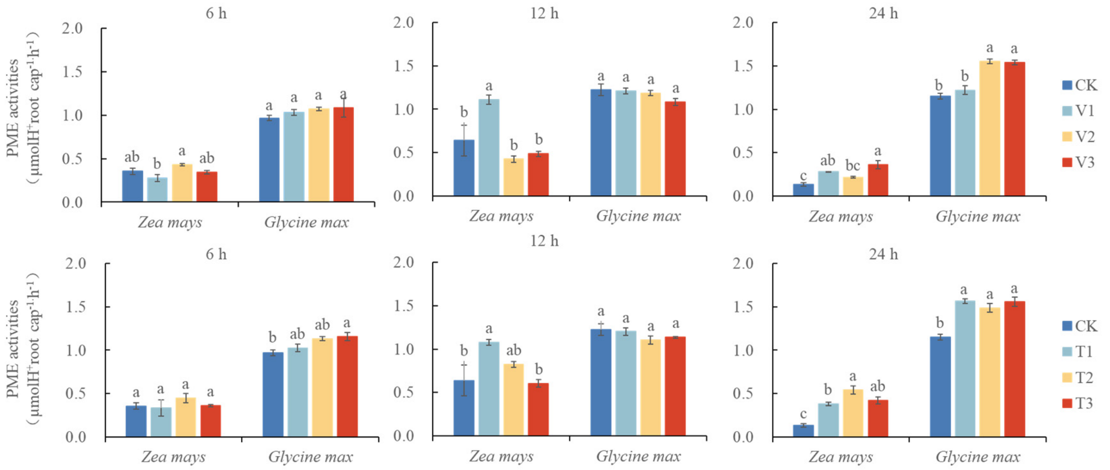

Figure S10. Pectin methylase(PME) activity

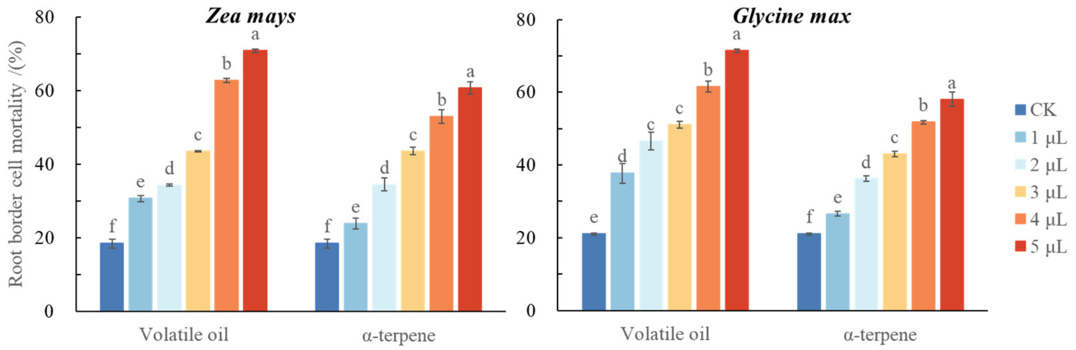

Figure S11. Changes in the activity of receptor root border cells under the action of volatiles from *C. ambrosioides*
